# Application of Bld-1-Embedded Elastin-Like Polypeptides in Tumor Targeting

**DOI:** 10.1038/s41598-018-21910-z

**Published:** 2018-03-01

**Authors:** Vijaya Sarangthem, Eun A. Cho, Aena Yi, Sang Kyoon Kim, Byung-Heon Lee, Rang-Woon Park

**Affiliations:** 1 Department of Biochemistry and Cell Biology, Cell & Matrix Research Institute, Kyungpook National University, School of Medicine, Daegu, 41944 Republic of Korea; 2 Laboratory Animal Center, Daegu-Gyeonbuk Medical Innovation Foundation, Daegu, Republic of Korea

## Abstract

Expression of various molecules on the surface of cancer cells compared to normal cells creates a platform for the generation of various drug vehicles for targeted therapy. Multiple interactions between ligands and their receptors mediated by targeting peptide-modified polymer could enable simultaneous delivery of a drug selectively to target tumor cells, thus limiting side effects resulting from non-specific drug delivery. In this study, we synthesized a novel tumor targeting system by using two key elements: (1) Bld-1 peptide (SNRDARRC), a recently reported bladder tumor targeting peptide identified by using a phage-displayed peptide library, and (2) ELP, a thermally responsive polypeptide. B_5_V_60_ containing five Bld-1 peptides and non-targeted ELP_77_ with a thermal phase-transition over 37 °C were analyzed to determine their bioactivities. Further studies confirmed the superior binding ability of B_5_V_60_ to bladder tumor cells and the cellular accumulation of B_5_V_60_ in cancer cells was dependent on the expression level of sialyl-Tn antigen (STn), a tumor-associated carbohydrate antigen. Additionally, B_5_V_60_ displayed excellent localization in bladder tumor xenograft mice after intravenous injection and was strictly confined to sialyl-Tn antigen-overexpressing tumor tissue. Thus, our newly designed B_5_V_60_ showed high potential as a novel carrier for STn-specific targeted cancer therapy or other therapeutic applications.

## Introduction

Delivery of drugs to tumor sites is necessary for effective cancer treatment. Therapeutic drug levels within a tumor are reduced due to the complex physiology and morphology of tumor tissue. Moreover, some chemo- and radiotherapeutic agents are toxic and cause undesirable side effects in healthy cells. Targeted-based delivery of drugs to tumors can improve cancer therapies by allowing anticancer agents to accumulate directly in tumor tissues, thereby enhancing overall therapeutic efficacy while minimizing systematic toxicity and side effects resulting from non-specific delivery of drugs^1^. Many active targeting strategies have been explored based on the unique features of tumors, including overexpressed receptors^2,3^, up-regulated enzymes^4,5^, reduced pH^6,7^, and hypoxia^8,9^ to enhance systematic delivery of drug carriers to tumors. Tumor cells have many molecular markers distinct from normal cells, such as high expression of growth factor receptors and variable expression of integrins, especially those that transmit growth signals^10,11^. The multivalent display of these targeting moieties on the exterior of nanoparticles or macromolecular carriers such as polymeric micelles, liposomes, proteins, and synthetic macromolecular polymers can result in increased avidity towards their corresponding receptors, which are overexpressed on tumor cells^12–14^. Moreover, studies have shown that theranostic agents based on nanovectors (liposomes, nanosomes, polymeric micelles) externally modified with targeting peptides are able to simultaneously carry a drug and contrast agent to target cells with an imaging probe co-incorporated in order to monitor therapy^15^.

Many peptides targeting specific organs, tumors, or proteins have been identified by the phage display screening method^16–19^. The three amino acid sequence RGD is a noble example of a targeting peptide that binds specifically to tumor vascular endothelial cells and also inhibits tumor angiogenesis^20,21^. Recently, Lee *et al*. identified a small peptide, SNRDARR, referred to as Bld-1, which shows high binding affinity to human bladder tumor tissue along with negligible binding to normal tissue^22^. Further study revealed that Bld-1 peptide is useful in detecting tumor cells in urine based on its weak binding to urinary cells of patients showing inflammation or to healthy individuals’ cells^23^. Additionally, Bld-1 peptide shows homology with SIRDARR motif in human sialic acid-binding immunoglobulin-like lectins 6 and 9 (Siglec 6 and 9), which interact with Neu5Acα2–6GalNAcα1(sialyl-Tn abbreviated as STn), a tumor-associated carbohydrate antigen that is overexpressed in various tumor cells^24–26^. Overexpression of these antigens has been correlated with cancer progression, poor prognosis, and an immunosuppressive microenvironment, which suggests they are important therapeutic targets. So far therapeutic vaccination used for clinical trials and antibodies against this antigen have limited success due to low immunogenicity or specificity, thereby development of innovative targeted delivery system is needed for effective cancer treatment.

To create a targeted system with multiple targeting sites, we can exploit thermally responsive recombinant elastin-like polypeptide (ELP), as it can be readily tailored with desirable biological and mechanical properties. ELPs consist of Val-Pro-Gly-Xaa-Gly pentapeptide repeats (with “guest residue” Xaa is any amino acid except Pro) derived from a structural motif found in mammalian elastin^27^. Compared to other polymeric drug delivery systems, ELPs are biodegradable, biocompatible, and less toxic^28,29^. ELPs undergo an inverse temperature phase transition, in which they are soluble at temperatures below their transition temperature (Tt) but become insoluble and aggregate at temperatures above their Tt^29^. ELP polymers can be synthesized at the genetic level by recombinant DNA methods, which means that their sequences, compositions, and molecular weights can be precisely tuned. ELP polymers can be easily expressed and purified at high yield simply by exploiting the inverse temperature cycling (ITC) method^27^. Studies have shown that genetically encoded synthesis of ELPs can be used to specify the location at which a biological drug, peptide, or protein is attached to an ELP sequence. Functionalization of ELP with targeting and internalization peptides has been found to improve accumulation and intracellular delivery of drugs at disease sites^30^. Fusion of ELP with a cell-penetrating peptide such as peptide derived from Drosophila Antennapaedia transcription factor (penetratin), HIV transactivation of transcription (TAT), and Kaposi fibroblast growth factor signal peptide (MTS) could increase intracellular delivery towards therapeutic targets, thereby enhancing drug efficacy^30^. Recently, it was demonstrated that penetratin-functionalized ELP-based delivery of kinase inhibitor peptide p21 induces enhanced cancer cell death. ELP diblock copolymers designed with cell-penetrating peptide (CPP) domain at the hydrophilic end and a therapeutic domain at the hydrophobic end form a thermally responsive micelle-like structure and induce the multivalent display of CPP on the exterior to enhance cellular internalization^31^.

In a previous study, AP1-ELP polymers containing multiple IL-4 receptor-targeting peptides were shown to increase intracellular localization into tumor tissue^32^. Multivalent display of targeting peptide AP1 along the backbone of ELP polymer increased affinity towards its target, IL-4 receptor, by approximately 10,000-fold compared to free peptides. We performed further studies to create another multivalent targeted based ELP, containing bladder tumors targeting peptide Bld-1. Thus, to improve the binding avidity and specificity of Bld-1, B_5_V_60_ was prepared by introducing multivalent Bld-1 into ELP polymer by genetic engineering, after which it’s physical and bio-chemical properties were analyzed. Tumor targeting activities of B_5_V_60_ were examined both *in vitro* and *in vivo*. Notably, the correlation of STn expression and B_5_V_60_ binding with cancer cells was investigated. Competitive inhibition of cell binding induced by B_5_V_60_ towards anti-STn antibody could be useful in STn-based targeting of cancer cells as a drug delivery system as well as in enhancing immune responses against immunotolerant tumor cells highly expressing STn.

## Results and Discussion

### Design and Preparation of Bld-1 ELP

**Figure 1:**
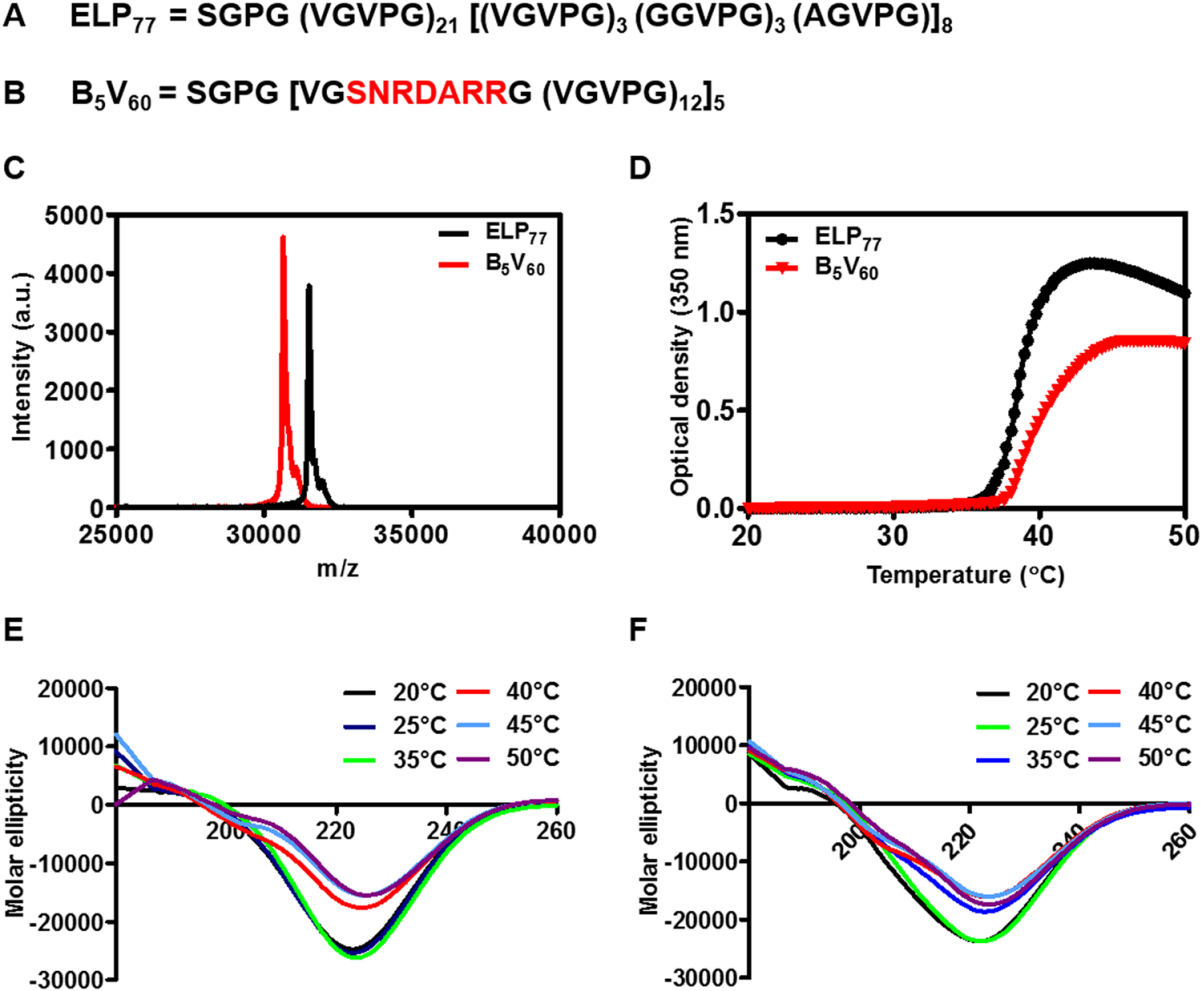
Chemical characterization of B_5_V_60_ and ELP_77_. Corresponding amino acid sequences of ELP_77_ (**A**) and B_5_V_60_ (**B**). (**C**) MALDI-TOF MS spectra of B_5_V_60_ and ELP_77_. (**D**) Turbidity profiles of B_5_V_60_ and ELP_77_ proteins were monitored by measuring the absorbance at 350 nm at a rate of 1 °C/min. Secondary structure of proteins (**E**) ELP_77_ and (**F**) B_5_V_60_ was analyzed using Circular Dichroism at different temperatures and 25 *µ*M concentration.

As ELPs can be synthesized at the genetic level by the recombinant DNA method, their sequences, compositions, and molecular weights can be precisely controlled^27^. In addition, ELP polymer can accommodate any target-specific ligands or functional groups as well as support multivalent presentation without any physiological changes or biological activities. Accordingly, in this study, we modified the coding sequence of ELP (VGVPG; with guest residue Valine) by incorporating bladder tumor-specific peptide (Bld-1; SNRDARR). The monomer gene referred to as B_1_V_12_ was designed with one Bld-1 sequence (SNRDARR) in its N-terminal region, followed by 12 pentapeptide repeats of ELP. Using the recursive directional ligation method, targeted polymer of variable lengths [B_1_V_12_]n were generated with periodic repetition of Bld-1 throughout the polypeptide sequences. The pentapeptide ELP sequence VGVPG was used to construct the Bld-1 ELP library. Since Valine is a hydrophobic guest residue with a low Tt, it is expected that the increase in Tt due to incorporation of hydrophilic Bld-1 peptide is moderated and maintained within a relevant temperature range suitable for clinical application. The control ELP was constructed by ligation of V_21_ with [V_3_ G_3_ A_3_]_8_ (Fig. 1A, Figure S1). Bld-1 ELP containing five Bld-1 peptides (B_5_V_60_) was used as a targeted polymer (Fig. 1B, Figure S1). V_21_-(V_3_G_4_A)_8_ referred to as ELP_77_ was used as a non-targeted polymer for further *in vitro* and *in vivo* experiments. B_5_V_60_ and control ELP_77_ proteins were expressed by IPTG induction and purified using the inverse transition cycling (ITC) method. After four rounds of ITC, B_5_V_60_ and ELP_77_ were analyzed by SDS-PAGE (Figure S2). The sizes of B_5_V_60_ and ELP_77_ were approximately ~30 kDa. Determination of accurate molecular weight by MALDI-TOF/MS confirmed the protein sizes of ELP_77_ (31341.8 Da) and B_5_V_60_ (30649.2 Da) (Fig. 1C).

### Thermal and Secondary Structure Characterization

Transition temperatures (Tt) of B_5_V_60_ and ELP_77_ proteins were monitored by measuring optical density at 350 nm as a function of temperature with 1 °C/min increments. Transition temperature (Tt) of ELP protein was defined as the temperature at 50% of the maximum of ELP aggregation. The Tt of B_5_V_60_ was in range of 37~40 °C (Fig. 1D). Due to the presence of charge residues in Bld-1 peptide, the Tt of B_5_V_60_ was elevated nearly 11 °C compared to the control ELP without targeting peptide. The Tt of B_5_V_60_ protein (39.77 °C) was just higher than physiological body temperature, whereas the same ELP without targeting peptide (negative control) had a Tt of ~28 °C (data not shown). Substitution of hydrophilic amino acids such as Glycine and Alanine at the fourth guest residue of the ELP pentapeptide repeat in the new non-targeted control ELP_77_ further increased Tt up to 38.67 °C (Fig. 1D), approximately similar to that of targeted B_5_V_60_. Measurement of turbidity profile at different concentration clearly reveal the dependency of Tt according to concentration (Figure S4). Despite lowering of Tt with increased concentration, their applicability was considered to be unaffected since relatively small change in Tt obtained by large increase in concentration^33^. Later determination of particle size at different temperatures (24, 37, and 50 °C) using DLS revealed the size increment with increased temperature in consistence with turbidity profile. But at physiological body temperature the size of ELP _77_ and B_5_V_60_ were 415.5 nm and 413.4 nm respectively (Figure S3). As tumor vessels are predicted to be leakier due to irregular development of vasculature and uncontrolled angiogenesis with pores ranging in size from 200 nm to 2 µm, thus both polypeptides will be well penetrable in tumor tissue^34^. Further, circular dichroism (CD) spectra confirmed changes in secondary structure along with an increase in temperature in both ELP_77_ (Fig. 1E) and B_5_V_60_ (Fig. 1F) respectively. Both polypeptides appeared to structurally consist of a helix and ß-turn in a random coil conformation. Helix content increased at higher temperature in B_5_V_60_ (Table S2), whereas helix formation decreased in ELP_77_ (Table S1). Both polymers displayed an increase in ß-turn content and slight decrease in random coil content at higher temperature. The increase in helix content in B_5_V_60_ was due to the presence of ligands Bld-1. However, incorporation of targeting ligands did not change the physical and chemical properties of ELP, clearly indicating its versatility in accommodating any functional peptide or protein.

### *In vitro* Cell Binding Analysis

**Figure 2:**
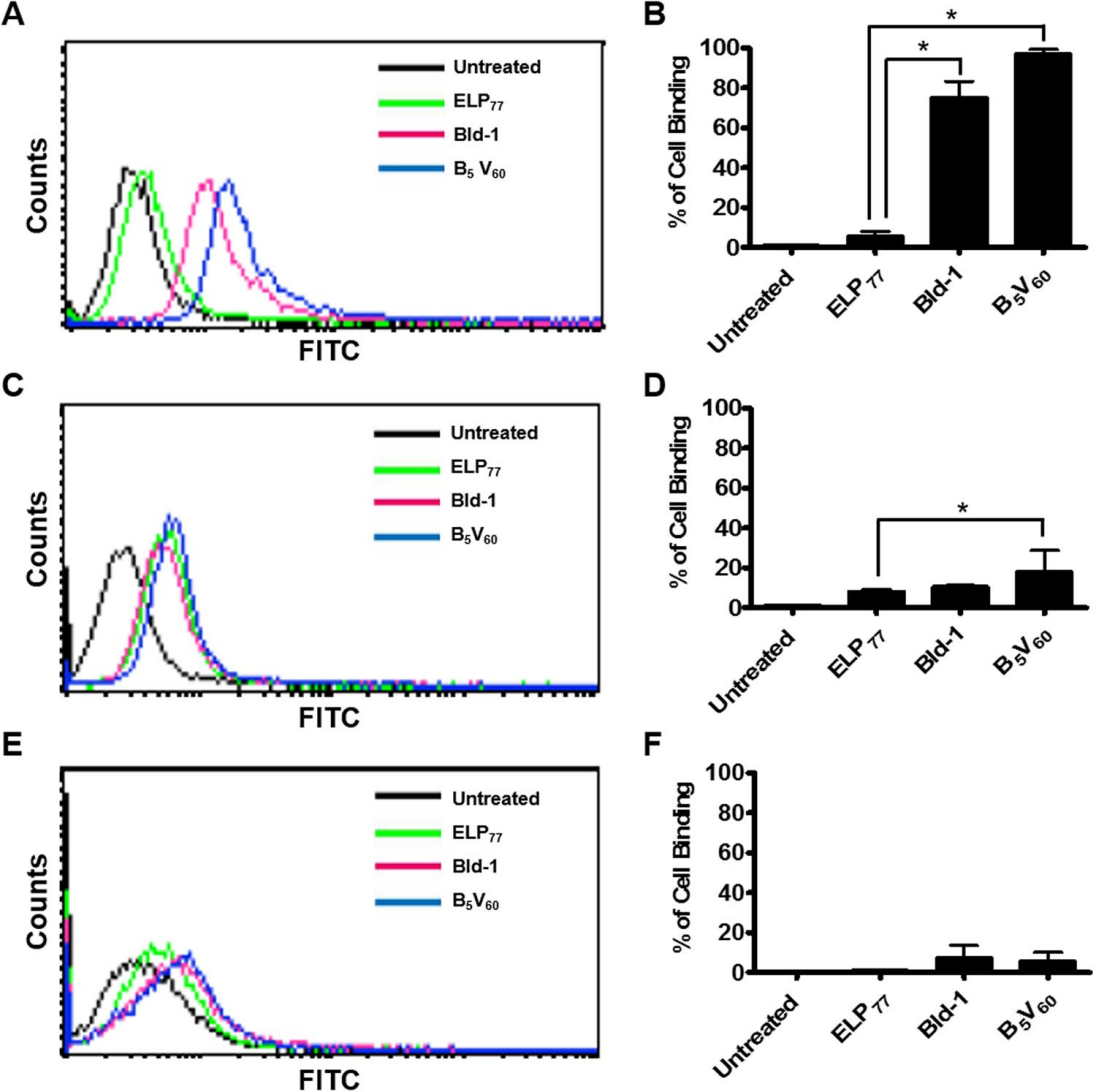
Determination of binding activity *in vitro*. 5637 (**A**,**B**), HT-29 (**C**,**D**), and HEK-293 (**E**,**F**) cells were incubated with 10 *µ*M B_5_V_60_ and ELP_77_ for 1 h at 4 °C. Percentage of cell binding was determined using flow cytometry. Histograms (on right) are representative of five independent experiments (n = 5). *P < 0.05 (Student’s t-test) when B_5_V_60_ and Bld-1 treated cells were compared to ELP_77_.

**Figure 3:**
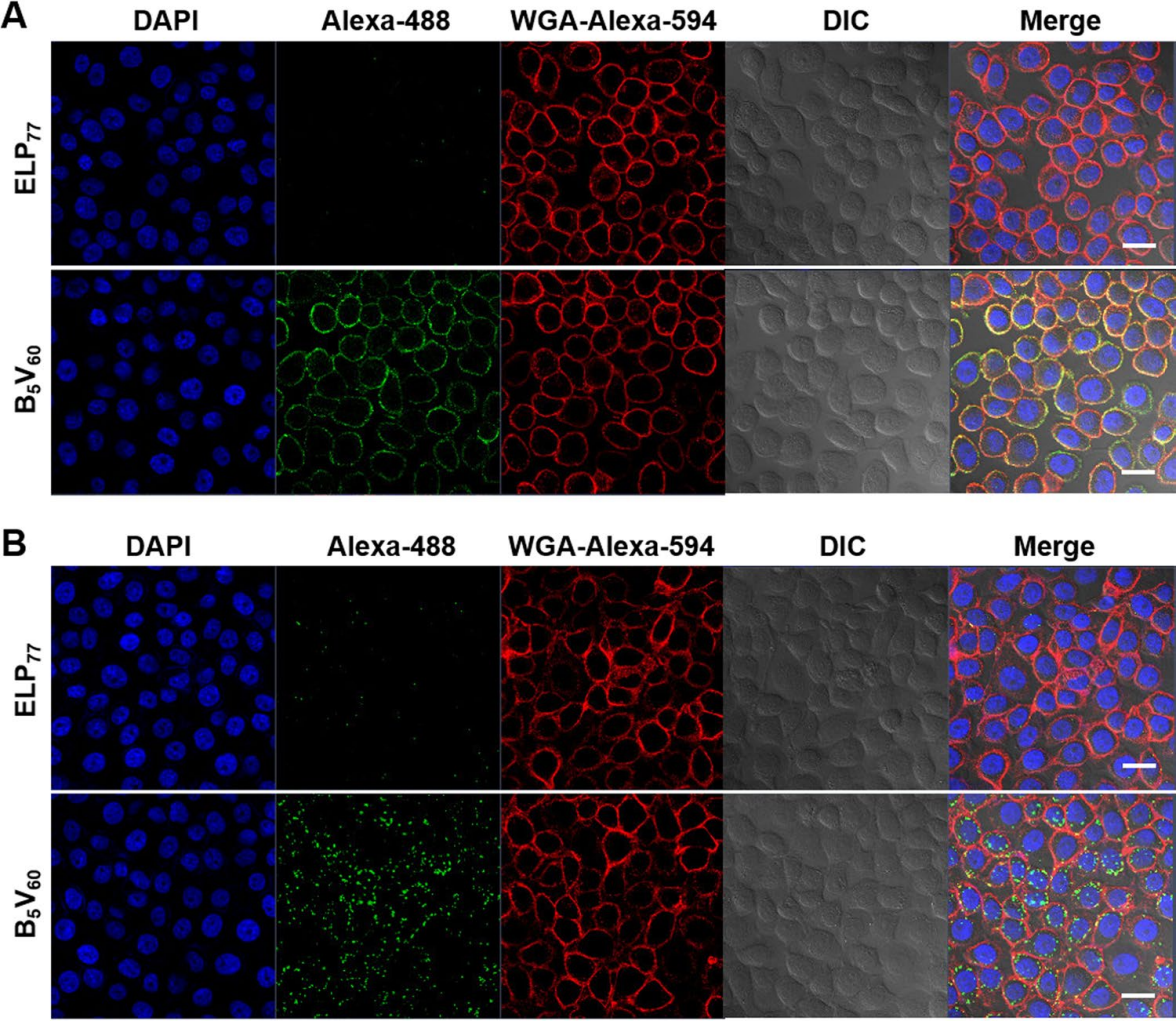
Studies of cellular localization *in vitro*. Confocal images of 5637 cells treated with 10 *µ*M B_5_V_60_ or ELP_77_ at 4 °C (**A**) and 37 °C (**B**). Cell membrane and nuclei were stained with WGA Alexa 594 and DAPI respectively. Representative confocal images of three experiments (Scale bar 20 *µ*m).

To analyze cell binding activity, Alexa 488-labeled ELP_77_ and B_5_V_60_ proteins were incubated with 5637, HT-29, and HEK293 cells, and cellular binding was accessed using flow cytometry. Targeted B_5_V_60_ polymer clearly revealed higher cellular binding activity compared to non-targeted ELP_77_ polymer after 1 h of incubation at 4 °C (Fig. 2A). B_5_V_60_ polymer showed 17.44 ± 2.08-fold greater cell binding activity compared to ELP_77_ in 5637 cells. Further, B_5_V_60_ polymer showed 1.29 ± 1.08-fold higher cell binding activity than free Bld-1 peptide (Fig. 2B). On the other hand, both polymers showed minimum binding activity in HT-29 (Fig. 2C,D) and HEK-293 cells (Fig. 2E,F). Thus, multivalent presentation of Bld-1 peptide along the ELP backbone increased cellular accumulation of tumor cells. In addition, B_5_V_60_ polymer showed 5.18 ± 1.28-fold greater cell attachment in 5637 cells compared to HT-26 cells. These results clearly suggest that B_5_V_60_ is highly specific to bladder tumors. Consistent with the flow cytometry data, confocal microscopy of adherent cells demonstrated that B_5_V_60_ polymer was localized more efficiently onto the surface of 5637 cells (Fig. 3A) at 4 °C. In contrast, minimum localization was observed in HT-29 (Figure S5A) and HEK293 (Figure S6A) cells upon incubation with respective polymers. Further, B_5_V_60_ polymer displayed improved cellular uptake towards 5637 cells (Fig. 3B) compared to HT-29 (Figure S5B) and HEK-293 (Figure S6B) cells upon incubation at 37 °C for 30 min. Neither ELP_77_ nor B_5_V_60_ showed significant cellular uptake by HT-29 and HEK-293 cells. These results clearly indicate that multiple Bld-1 peptide incorporation by the ELP polymer backbone resulted in greater tumor specific accumulation and uptake compared to the non-targeted ELP control. Together, it was confirmed that B_5_V_60_ showed no accumulation in normal cells, which is crucial for drug delivery systems.

### STn Expression Determination and Competition Assay

**Figure 4:**
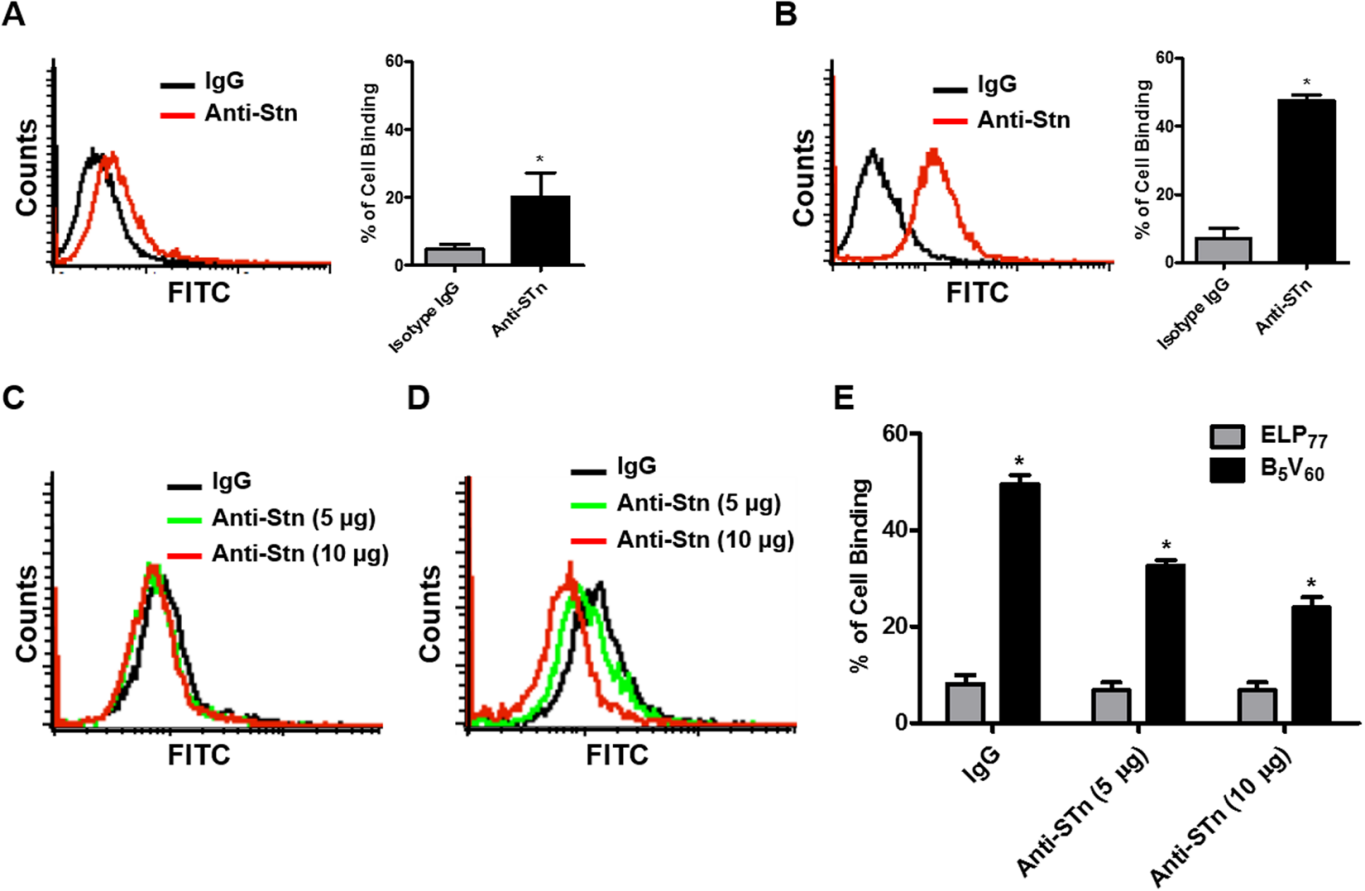
Estimation of STn expression and competition assay. STn expression levels of HT-29 cells (**A**) and 5637 cells (**B**) were measured using flow cytometry after incubation with anti-STn –Alexa 488 antibody for 1 h at room temperature. 5637 cells (1 × 10^6^) were pre-incubated with different concentrations of anti-STn antibody (5 and 10 *µ*g/ml) for 1 h at room temperature. The cells were further incubated with 10 *µ*M ELP_77_ (**C**) and B_5_V_60_ (**D**) at 4 °C. Histograms are representative of five independent experiments (n = 5). Inhibition of binding activity were analyzed through flow cytometry. (**E**) Percentage of cell binding by ELP_77_ and B_5_V_60_ after pre-incubation with anti-STn antibody. *P < 0.05 (Student’s t-test) for ELP_77_ versus B_5_V_60_.

In previous study it was specified through homology search that Bld-1 peptide shows similarity with SIRDARR motif found in human sialic acid binding immunoglobulin-like lectin 6 and 9 (Siglec 6 and 9) which interact with Neu5Aca2-6GalNAca1 (sialosyl-Tn, STn) a tumor-associated carbohydrate antigen, overexpressed in various tumor cells^24–26^. Thus, we investigated the level of STn expression in 5637 and HT-29 cells by flow cytometry. Higher expression (up to 45 ± 5%) of STn was observed in 5637 cells (Fig. 4B) in contrast to lower expression (around 15 ± 3%) in HT-29 cells (Fig. 4A). Increased binding of B_5_V_60_ to 5637 cells as well as lower binding to HT-29 cells may be correlated with STn expression. To confirm STn-dependent binding of B_5_V_60_, competition assay was performed where 5637 cells were pre-incubated with different concentrations of anti-STn antibody (5 and 10 *µ*g), and binding of respective polypeptide was measured by flow cytometry. Binding of B_5_V_60_ was remarkably reduced in a concentration-dependent manner upon pre-incubation with anti-STn antibody (Fig. 4D,E). At a higher antibody concentration of 10 *µ*g, binding of B_5_V_60_ was reduced by two-fold in comparison with the isotype control. Minimum or no change in binding was observed when cells were incubated with ELP_77_ (Fig. 4C,E). This result demonstrates that accumulation of B_5_V_60_ on cells is highly dependent on the level of STn expression by cancer cells.

**Figure 5:**
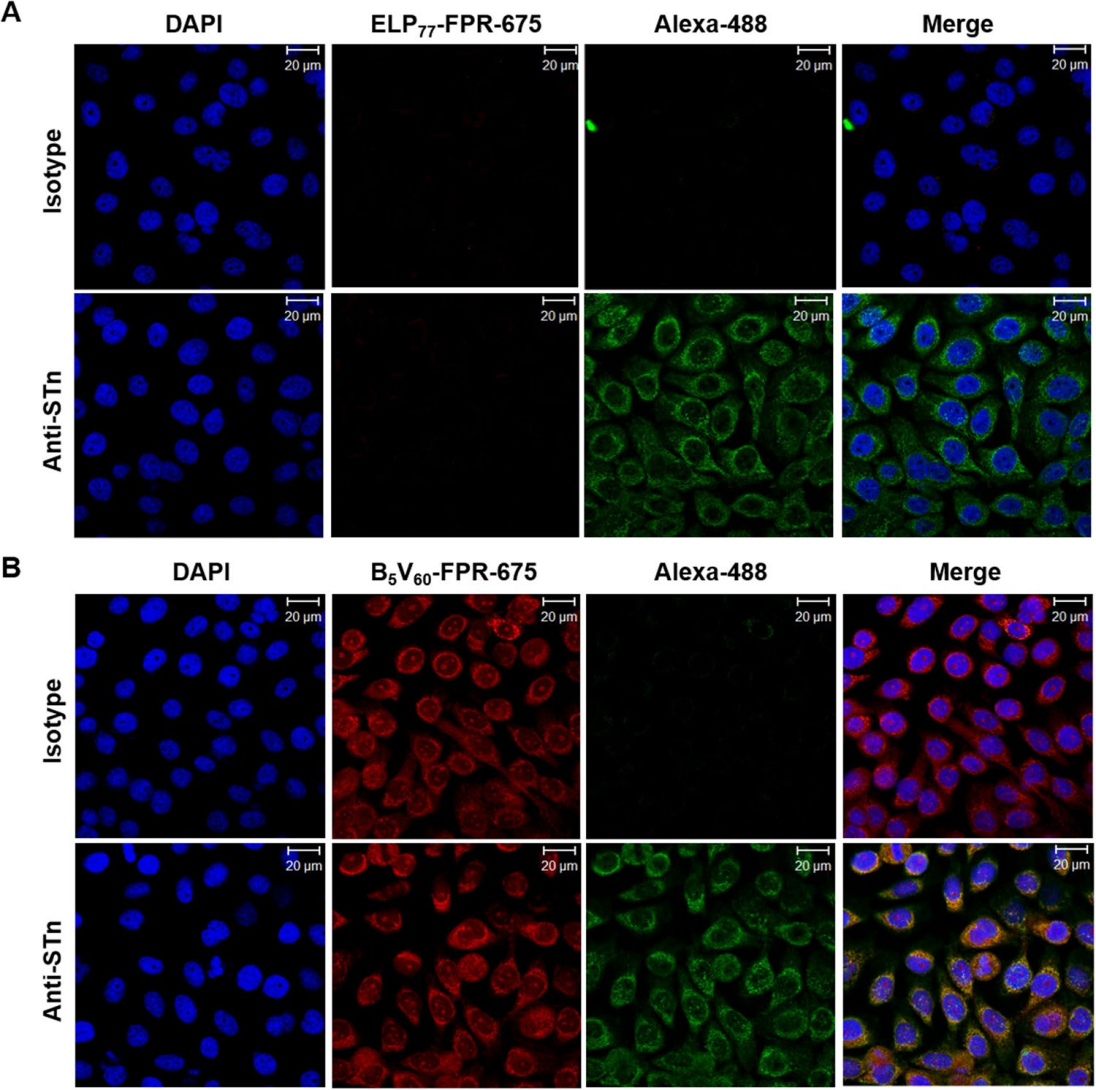
Co-localization Assay. 5637 cells (1 × 10^6^) were pre-incubated with anti-STn-Alexa 488 (1:100) and further incubated with 10 *µ*M ELP_77_ (**A**) and B_5_V_60_ (**B**) at 4 °C. Co-localization of protein and antibody was analyzed using confocal microscopy. Representative confocal images of three experiments (Scale bar 20 *µ*m).

Next, we performed co-localization assay to confirm specific B_5_V_60_ binding to STn, which is highly expressed in some tumor cells. Confocal microscopic images clearly revealed a greater accumulation of B_5_V_60_ and anti-STn antibody on the surface of 5637 cells (Fig. 5B). Minimum accumulation was observed when cells were incubated with ELP_77_ (Fig. 5A). Merged image showed improved co-localization, which further proves that both proteins shared the same binding target. On the other hand, B_5_V_60_ and anti-STn antibody displayed lower binding in HT-29 cells (Figure S7). These results demonstrate that binding of B_5_V_60_ to tumor cells is dependent on STn expression by cancer cells.

### *In vivo* Biodistribution of B_5_V_60_ in Tumor Bearing Mice

**Figure 6:**
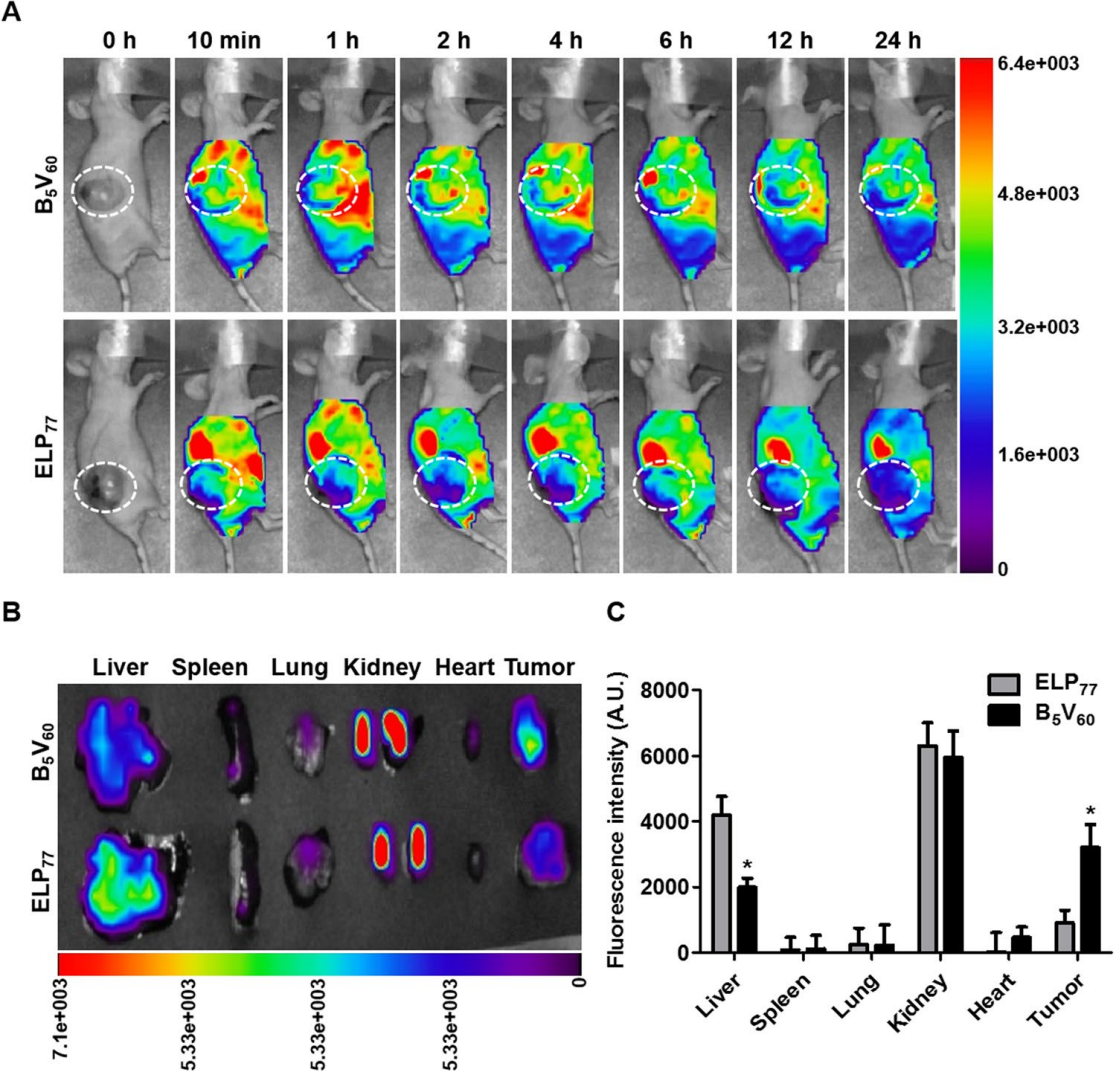
*In vivo* imaging and biodistribution of B_5_V_60_. **(A**) ELP_77_ and B_5_V_60_ labeled with FNR 675 were intravenously injected into 5637 tumor xenograft nude mice. Fluorescence images were taken at different time intervals such as 0.1, 1.2, 4, 6, 12, and 24 h to study biodistribution *in vivo* (n = 10). Scale bar indicates normalized fluorescence intensity. (**B**) Fluorescence images of excised organs and tumor at 24 h after intravenous injection. Representative images of subsequent 10 experiments. Scale bar indicates normalized fluorescence intensity. (**C**) Analysis of fluorescence intensities of excised organs, including tumor tissue (n = 8). *P < 0.05 (Student’s t-test) for ELP_77_ versus B_5_V_60_.

**Figure 7:**
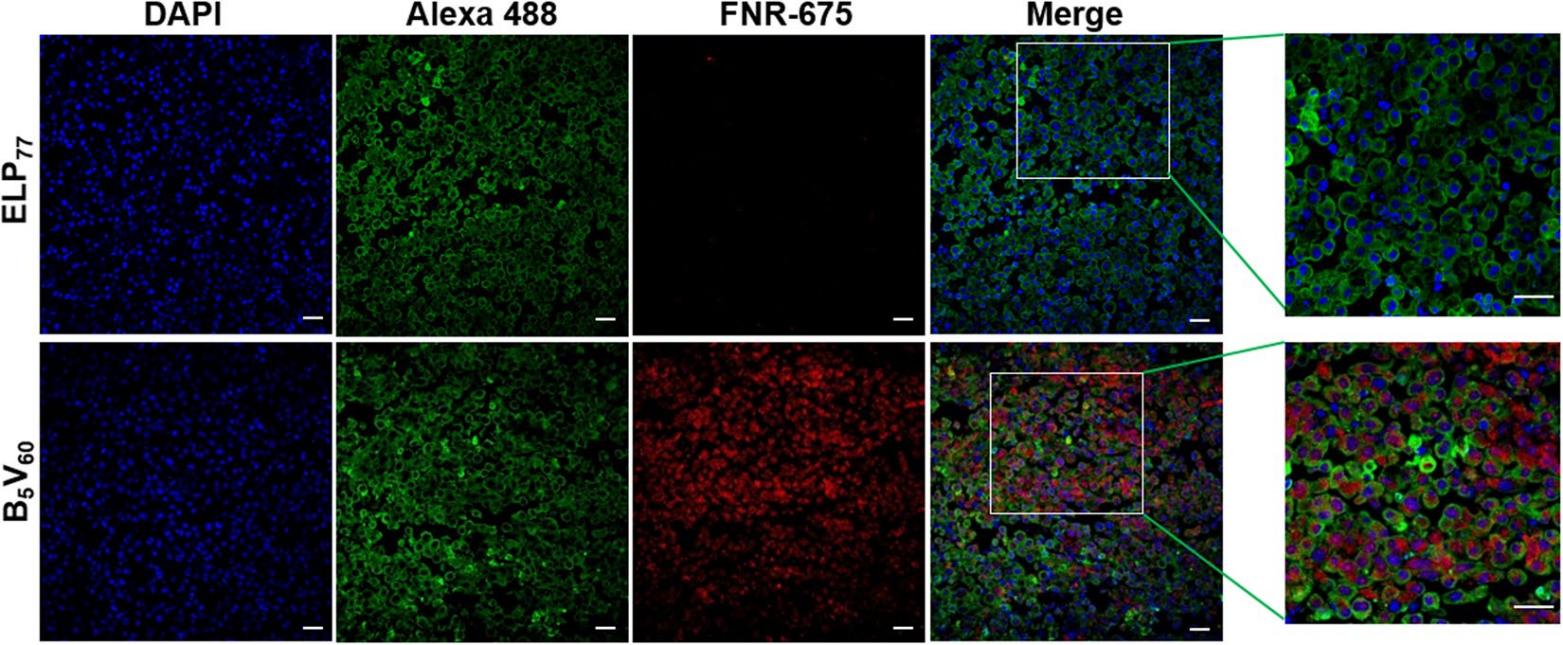
*Ex vivo* staining: Cryo-section of tumor tissue stained with anti-STn and observed by confocal microscopy. Blue-nuclei stained with DAPI, Green- tumor cells stained with anti-STn-Alexa 488, Red-polypeptide labeled with FNR 675. (Scale bar 20 *µ*m).

In relation to the significant tumor-specific *in vitro* targeting activity of B_5_V_60_, *in vivo* selective homing towards tumor tissue was determined using a live optical imaging system. Prior to the biodistribution study, the stabilities of FPR-675-labeled polypeptides were examined by incubation in fresh plasma for different time intervals at 37 °C. Measurement of fluorescent intensities with excitation and emission wavelengths of 675 nm and 698 nm, respectively, after ITC confirmed that nearly 18–20% of dye was released over 24 h, confirming the dye was firmly conjugated to its respective polypeptides (Figure S8A). Further measurement of the fluorescent intensities of serially diluted labeled B_5_V_60_ and E_147_ polypeptides showed a dose-dependent decrease in intensity with no significant difference (Figure S8B). Thus, after confirming labeling efficiency, athymic mice bearing 5637 bladder tumors were injected intravenously with respective polymers labeled with FNR-675. Near infrared fluorescence (NIRF) images taken at different time intervals showed that B_5_V_60_ polymers were rapidly distributed within 10 min and accumulated time-dependently in tumor tissue (Fig. 6A). At 2 h post-injection, high fluorescence intensity in target tumors was observed in mice injected with B_5_V_60_ polymer and persisted longer than 24 h (Figure S9). In contrast, the ELP_77_ control showed low accumulation towards tumor tissue compared to high accumulation in other organs. Thus, despite having the same chemical characteristics, B_5_V_60_ showed superior tumor accumulation than the ELP_77_ control due to the presence of multiples copies of targeting ligands. *Ex vivo* fluorescence images of excised tumors and organs collected at 24 h post-injection showed a 2.3-fold increase in fluorescence intensity in target tumors of B_5_V_60_-injected mice compared to ELP_77_ injection (Fig. 6B). Higher accumulation in kidneys was observed in both ELP_77_ and B_5_V_60_-injected mice due to rapid metabolism. Since protein molecular weight has a strong effect on biodistribution *in vivo*, higher kidney accumulation may be attributed to the lower molecular weights of both polypeptides^35^. However, fluorescence intensity in the liver was stronger in ELP_77_-injected mice compared to B_5_V_60_ (Fig. 6C). Together, immunohistological examination of tumor tissue showed that B_5_V_60_ was highly confined to STn-expressing tumor tissue, consistent with *in vivo* and *ex vivo* imaging results (Fig. 7). Collectively, these results further confirm the potential of B_5_V_60_ as a candidate macromolecular drug carrier for cancer therapy.

## Conclusion

In this study, we demonstrated the potential of genetically encoded synthesis of ELP polypeptide in which multiple tumors targeting peptides are randomly incorporated into the polypeptide backbone without the need for covalent attachment chemistry. Multivalent presentation of bladder tumor-targeting peptide onto the ELP backbone can increase cellular uptake compared to monovalent Bld-1 peptide and the non-targeted ELP control. Consistent with previous findings demonstrating the multivalent presentation of ELP with IL-4 receptor-targeting peptide has efficient targeting ability both *in vitro* and *in vivo*, this study further confirms our strategy of seamlessly incorporating functional peptides. An *in vivo* study revealed that B_5_V_60_ polymer accumulated in tumor tissue and was retained for over 24 h. Accumulation of B_5_V_60_ on tumor cells was highly correlated with the expression level of STn (sialyl-Tn). Since high expression of STn on cell surfaces is related with advanced-stage tumor and malignancy, our newly design B_5_V_60_ polymer offers an approach for application of novel therapeutics such as selective drug-delivery or STn-based immunotherapy. Subsequently, this strategy can be further optimized to study the association of tumor-associated carbohydrate antigens with immune cells.

## Methods

### ELP Nomenclature

ELPs are designated as ELP [X_a_Y_b_Z_c_]n where X, Y, and Z specify the guest residue, a, b, and c are the numbers of corresponding guest residue repeats, and ‘n’ denotes the number of monomer gene repeats for RDL. For example, V_3_G_4_A consists of seven pentapeptide XGVPG repeats with Valine, Glycine, and Alanine as guest residues (X). In this experiment, B_5_V_60_ consisted of B as Bld-1 peptide and V as VGVPG with 60 repeats. [V_21_ (V_3_G_3_A)_8_] refers to an ELP_77_ consisting of 77 pentapeptide repeats with Valine, Glycine, and Alanine as guest residues (VGVPG, GGVPG, AGVPG) used as a control.

### ELP Gene Oligomerization and Expression

Synthetic oligonucleotides encoding monomer genes of V_7_, (V_3_ G_3_ A) and VGSNRDARRG-V_5_ containing *Bam*H I, *Pfl*M I, *Bgl* I, and *Hin*D III enzyme sites were obtained from Macrogen Inc. Seoul, Korea. Corresponding oligonucleotides were annealed and ligated into *Bam*H I and *Hin*D III double-digested pRSET B vector. The pRSET B containing V_7_ was linearized with *Pfl*M I, enzymatically dephosphorylated with Calf intestinal alkaline phosphatase (CIP), and ligated with VGSNRDARRG-V_5_ double-digested with *Pfl*M I and *Bgl* I. The resulting (VGSNRDARRG-V_5_)_2_ designated as B_1_V_12_ was used as a monomer gene to synthesize Bld-1 ELPs with various lengths by RDL. On the other hand, V_7_ and V_3_G_4_A were used as monomer genes to synthesize ELPs with various length by RDL. After gene oligomerization by RDL, Bld1-ELP with various lengths (n = 2, 4, 5, and 6) and different transition temperatures was obtained, and B_5_V_60_ containing five Bld-1 peptides was used for further experimentation. In order to construct ELP_77_ control [V_21_ (V_3_G_4_A)_8_], pRSET B containing V_21_ was doubled-digested with *Pfl*M I and *Bgl* I and ligated into (V_3_G_3_A)_8_ containing vector linearized with *Pfl*M I. Positive colony were confirmed through restriction digestion with *Bam*H I and *Hin*D III, followed by gene sequencing (Macrogen Inc. Seoul, Korea).

### ELP Gene Expression

For protein expression, expression vector pET 25 b+ vector was modified by ligation with annealed oligonucleotides encoding sense 5′-TATGAGCGGGCCGGGCTGGCCGTGCTAAA-3′ and anti-sense 5′-AGCTTTTAGCACGGCCAGCCCGGCCCGCTCA-3′ containing *Nde* I, *Sfi* I, and *Hin*D III restriction enzyme sites. After confirmation through DNA sequencing, modified pET25b+ vector was digested with *Sfl* I, and ELP_77_ or B_5_V_60_ gene was ligated and transformed into DH5α competent *E. coli*. Positive colonies were confirmed through restriction digestion with *Nde* I and *Hin*D III, followed by gene sequencing (Macrogen Inc. Seoul, Korea).

### ELP Protein Purification

For protein expression, BL21 (DE3) chemically competent *E. coli* cells were further transformed with modified pET 25b+ vector containing ELP_77_ and B_5_V_60_ gene. Starter cultures were prepared after inoculation of expression strain in 15 ml of Circle Grow media supplemented with 100 *μ*g/ml of ampicillin overnight at 37 °C. The starter cultures were further incubated with 800 ml of fresh Circle Grow media containing ampicillin at 37 °C until it reached 0.8–1.0 at O.D 600. Protein expression was induced by addition of 1 mM solution of IPTG. Cells were then harvested after 4 h by centrifugation at 4000 rpm for 20 min at 4 °C and suspended in 10 ml PBS. Cells were lysed by sonication at 4 °C and purified using inverse transition cycling (ITC). Four rounds of ITC were conducted to remove all contaminants. ELP expression and purity were analyzed by SDS-PAGE, followed by Copper chlorite staining. Protein concentration was measured by Cary UV-Vis spectroscopy using an extinction coefficient of 5690 M^−1^ cm^−1^ for both ELP_77_ and B_5_V_60_.

### MALDI TOF/MS Analysis

Accurate molecular weights of ELP_77_ and B_5_V_60_ were determined using an UltrafleXtreme (Bruker). For the measurement, proteins were dissolved with 0.1% trifluoroacetic acid and mixed with an equal volume of matrix solution (1:1). Resultant mixture (1 *µ*l) was then applied to a standard steel target for drying at room temperature. The spectra were obtained after calibration with standards.

### Thermal Characterization

Transition temperature (Tt) of ELP_77_ and B_5_V_60_ were determined by monitoring the turbidity profiles of protein solutions at a wavelength of 350 nm as a function of temperature using a UV-visible spectrophotometer (Agilent Technologies, CA, USA). The absorbance was monitored from 20 °C to 50 °C with 1 °C/min increments. The first derivative of the turbidity profile with respect to temperature was numerically calculated, and the Tt was defined as the solution temperature at 50% of the maximum turbidity gradient. The Tt of ELP_77_ and B_5_V_60_ protein were monitored at 10 *μ*M concentration.

### Flow Cytometric Analysis

A total of 2 × 10^5^ HEK293 (Human Embryonic Kidney), HT-29 (Human Colorectal Adenocarcinoma) and 5637 (Human Bladder Carcinoma) cells were incubated with 10 *μ*M Alexa 488-labeled Bld-1 peptide, ELP_77_, and B_5_V_60_ protein for 1 h at 4 °C. Cells were washed twice with PBS and suspended in 200 *μ*l of PBS then subjected to flow cytometric analysis (BD Bioscience, San Jose, CA, USA). For analysis 20,000 cells were counted for each sample.

### Confocal Microscopy

To test binding specificity, 5637, HT-29, and HEK293 cells were seeded on a four-chambered slide (8 × 10^4^/well) and grown to 80% confluence. After 24 h, cells were incubated with 10 *μ*M Alexa 488-labeled ELP_77_ and B_5_V_60_ for 1 h at 4 °C or for 30 min at 37 °C. Cells were then fixed with 4% paraformaldehyde (Sigma Aldrich) and cell membrane marker, Wheat germ agglutinin (WGA) Alexa Fluor 594 conjugate (Molecular Probes, Inc., Eugene), and cell nuclei were stained with DAPI (Sigma Aldrich). Images were captured by a Zeiss LSM-510 Meta confocal microscope.

### STn Expression analysis

5637 and HT-29 (1 × 10^6^) cells were incubated with anti-Sialyl Tn antibody (Abcam, Seoul, South-Korea) and IgG isotype antibody as a control for 1 h at room temperature. Cells were further washed with PBS to remove excess or unbound antibody and subjected to flow cytometry to measure the level of STn receptor expression. A total of 20,000 events were collected for each sample.

### Competition Assay

A total of 1 × 10^6^ 5637 cells were collected and pretreated with anti-Sialyl Tn antibody at different concentrations (5 and 10 *µ*g) at room temperature for 1 h. Cells were washed and incubated with 10 *µ*M Alexa 488-labeled proteins for 1 h at 4 °C. After several washes with PBS, cells were suspended with 200 *µ*l of PBS and subjected to flow cytometry.

### Co-localization Assay

A total of 8 × 10^4^ 5637 and HT-29 cells were seeded on a four-chambered slide. After 24 h, cells were incubated for 1 h with anti-Sialyl Tn (1:100) antibody labeled with FITC at room temperature. Cells were further incubated with 10 *μ*M FNR 675-labeled E77 and B_5_V_60_ for 1 h at 4 °C. After several washes with PBS, cell nuclei were stained with DAPI (Sigma Aldrich) for 3 min. Images were captured by a Zeiss LSM-510 Meta confocal microscope.

### *In vivo* Fluorescence Imaging

All animal experiments were reviewed and approved by the Committee on the Ethics of Animal Experiments of the Kyungpook National University (Permit Number KNU 2016–0083). This study strictly followed the recommendations of National Institute of Health (NIH) for the Care and Use of Laboratory Animals. Athymic nude mice (BALB/c nu/nu) were housed in a specific pathogen-free environment at 22 ± 2 °C, 55 ± 5% relative humidity with light. Tumors were created by subcutaneously injecting 5637 cells (5 × 10^6^ cells) into the right flanks of 5 week-old female mice. Tumors size (3–5 mm in diameter) usually develop within 1 month. Actually, the tumor sizing around 5 mm in diameter is more effective for our peptide delivery studies rather than tumor size below 3 mm in diameter. Mice bearing a subcutaneous tumor were anesthetized with 1.5% isoflurane inhalation and injected intravenously with approximately 3 mg/kg of FNR675-labeled ELP_77_ (n = 10) and B_5_V_60_ (n = 10). *In vivo* fluorescence images were taken at different time points after anesthetization (0 min, 1 h, 2 h, 4 h, 6 h, 12 h, and 24 h) using Optix eXplore (ART, Advanced research technologies Inc., Montreal, Canada).

### *Ex vivo* Fluorescence Imaging and Tissue Preparation

Twenty-four hours after intravenous injection, animals were euthanized with CO_2_, and tumor and organs were collected. *Ex vivo* fluorescence images were then taken. Tumor tissues were fixed with 4% paraformaldehyde overnight and frozen for cryosectioning. Tissues were sectioned with 8 mm thickness and incubated with anti-STn antibody (1:100) overnight. Tissues were stained with Alexa 488-labeled goat anti-mouse IgG secondary antibody (1:200), whereas nuclei were stained with DAPI and observed under a confocal microscope.

### Statistical Analysis

Statistical significance of differences between experimental and control groups was analyzed by Student’s t-test for two groups or one–way analysis of variance (ANOVA) for more groups. P < 0.05 was set as statistical significance, was denoted by asterisks in the figures.

## Electronic supplementary material

Supplementary information
