# Supplementary material for: Application of Bld-1-Embedded Elastin-Like Polypeptides in Tumor Targeting
Source: Sci Rep. 2018 Mar 1;8:3892. doi: 10.1038/s41598-018-21910-z (PMC5832811; doi:10.1038/s41598-018-21910-z)
Supplement: Supplementary file 1 — Supplementary information [file 41598_2018_21910_MOESM1_ESM.docx]

**Application of Bld-1-Embedded Elastin-Like Polypeptides in Tumor Targeting**

Vijaya Sarangthem,^a,^**^+^** Eun A. Cho,^a,^**^+^** Aena Yi,^a^ Sang Kyoon Kim,^b^ Byung-Heon Lee,^a^ Rang-Woon Park^a,^*

^a^ Department of Biochemistry and Cell Biology, Cell & Matrix Research Institute, Kyungpook National University, School of Medicine, Daegu 41944, Republic of Korea.

^b^ Laboratory Animal Center, Daegu-Gyeonbuk Medical Innovation Foundation, Daegu, Republic of Korea

***Corresponding Author:**

Rang-Woon Park, M.D., Ph.D.

Department of Biochemistry and Cell Biology, School of Medicine,

Kyungpook National University, 101 Dongin-2ga, Jung-gu,

Daegu, 41944, Republic of Korea

E-mail: [nwpark@knu.ac.kr](mailto:nwpark@knu.ac.kr). Phone: +82 53 420 4822. Fax: +82 53 422 1466.

**^+^** Vijaya Sarangthem and Eun A. Cho equally contributed to this work

**S1. Methods**

**S1.1 Cell lines**

HEK293 (Human Embryonic Kidney), HT-29 (Human Colorectal Adenocarcinoma) and 5637 (Human Bladder Carcinoma) cells were obtained from the American Type Culture Collection (ATCC). 5637 and HT-29 cancer cells were grown in RPMI-1640 (High Clone, Invitrogen). HEK 293 cells were grown in Dulbecco’s modified Eagle’s medium (DMEM) medium (Sigma Aldrich, St Louis, MO, USA). All cells were cultured with 10% fetal bovine serum (Sigma Aldrich, St Louis, MO, USA) and 1% penicillin and streptomycin (PS, Sigma Aldrich). Cells were maintained at 37 °C in the presence of 5% CO_2_.

**S1.2 Labeling of ELP with Alexa 488**

B_5_V_60_ and ELP_77_ proteins diluted in PBS at a final concentration of 100 *μ*M were mixed with Sulfo-SMCC (succinimide 4-[N-maleimidomethyl] cyclohexane carboxylate) (Sigma Aldrich) at an equivalent molar concentration. Alexa Fluor 488-C5 maleimide (Invitrogen) dye dissolved in DMSO was added to the mixture at the same molar concentration as the protein. The reaction was maintained by constant rotation at 4 ºC overnight. Unexpected products were eliminated by dialysis (MWCO 6-8,000). Alexa 488-labeled polypeptides were separated from unreacted fluorophores by two rounds of ITC, concentrated to a total volume of 1 ml, and stored at -20 ºC. Degree of labeling was determined according to the Invitrogen conjugation manual.

**S1.3 Determination of labeled dye stability in plasma**

Prior to *in vivo* experiments the stability of conjugated dye was investigated. FPR 675-labeled ELP_77_ and B_5_V_60_ were incubated in fresh mouse plasma for various time intervals (1, 2, 4, 6, 12, and 24 h), and the protein was precipitated by using ITC. Fluorescence intensities of dye in the supernatant and pellet were measured at excitation and emission wavelengths of 675 nm and 698 nm, respectively, using a SpectraMax M plate reader (Molecular Devices, CA, USA). Percentage of released dye was calculated in reference with standard curve obtained from known quantities of labeled proteins.

**S1.4 Dynamic light Scattering**

The changes in particle size corresponding to different temperatures (24, 37, 50 ºCwas analyzed through DLS (ELS-Z2, Otsuka Electronics, Japan). Before analysis

protein solution (10 *µ*M) was filtered through Whatmann syringe filter with pore size 0.02 *µ*m. Hydrodynamic diameters were plotted using size by number distribution approach.

**S1.5 Thermal characterization**

Thermal characteristics (T_t_) of ELP_77_ and B_5_V_60_ were determined by measuring the optical densities of polypeptides at different concentration using a Cary UV-visible spectrophotometer equipped with a temperature controller (Agilent Technologies) at 350 nm. The absorbance was measured at temperatures ranging from 20 °C to 50 °C in 1°C min ^̶ 1^ increments.

**ELP_77_**

MSGPGVGVPGVGVPGVGVPGVGVPGVGVPGVGVPGVGVPGVGVPGVGVPGVGVPGVGVPGVGVPGVGVPGVGVPGVGVPGGGVPGGGVPGVGVPGAGVPGVGVPGGGVPGVGVPGGGVPGGGVPGVGVPGAGVPGVGVPGGGVPGVGVPGGGVPGGGVPGVGVPGAGVPGVGVPGGGVPGVGVPGGGVPGGGVPGVGVPGAGVPGVGVPGGGVPGVGVPGVGVPGVGVPGVGVPGVGVPGVGVPGVGVPGVGVPGVGVPGVGVPGVGVPGVGVPGVGVPGVGVPGVGVPGGGVPGGGVPGVGVPGAGVPGVGVPGGGVPGVGVPGGGVPGGGVPGVGVPGAGVPGVGVPGGGVPGVGVPGVGVPGVGVPGVGVPGVGVPGVGVPGVGVPGWPC

**B_5_V_60_**

MSGPGVGSNRDARRGVGVPGVGVPGVGVPGVGVPGVGVPGVGVPGVGVPGVGVPGVGVPGVGVPGVGVPGVGVPGVGSNRDARRGVGVPGVGVPGVGVPGVGVPGVGVPGVGVPGVGVPGVGVPGVGVPGVGVPGVGVPGVGVPGVGSNRDARRGVGVPGVGVPGVGVPGVGVPGVGVPGVGVPGVGVPGVGVPGVGVPGVGVPGVGVPGVGVPGVGSNRDARRGVGVPGVGVPGVGVPGVGVPGVGVPGVGVPGVGVPGVGVPGVGVPGVGVPGVGVPGVGVPGVGSNRDARRGVGVPGVGVPGVGVPGVGVPGVGVPGVGVPGVGVPGVGVPGVGVPGVGVPGVGVPGVGVPGWPC

**Figure S1.** Corresponding amino acid sequences of ELP_77_ and B_5_V_60_. Red: Bld-1 peptide incorporated periodically along with ELP sequences.

**
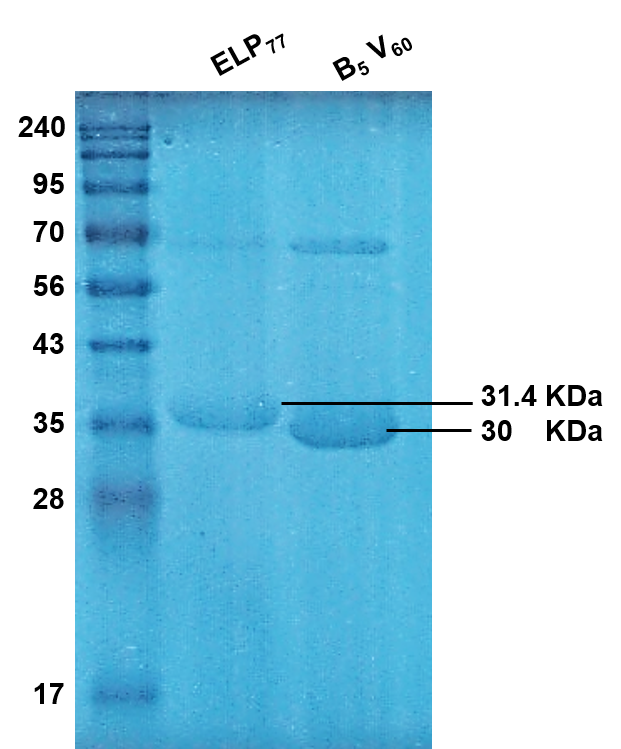
**

**Figure S2.** SDS-PAGE of expressed proteins visualized after copper staining. First lane: Standard protein marker in KDa, Second lane: ELP_77_ (31.4 KDa), Third lane: B_5_V_60_ (30 KDa). Upper band represents occurrence of dimer due to Cysteine residue present on C-termini of proteins.

**Table S1.** Secondary structural components ratio obtained from measured CD spectrum (residue molar ellipticity) of ELP_77_ at various temperatures.

|  |  | ELP_77_ |  |  |
| --- | --- | --- | --- | --- |
| Temperature | **Helix** | **Beta** | **Turn** | **Random** |
| 20°C | 31.4 | 0 | 18.2 | 50.3 |
| 25°C | 31.6 | 0 | 18 | 50.5 |
| 30°C | 32.8 | 0 | 18.1 | 49 |
| 35°C | 29.5 | 0 | 23.2 | 48.2 |
| 40°C | 28.9 | 0 | 25.6 | 45.5 |
| 45°C | 28.6 | 0 | 22.5 | 43.7 |

**Table S2.** Secondary structural components ratio obtained from measured CD spectrum (residue molar ellipticity) of B_5_V_60_ at various temperatures.

|  |  | B_5_V_60_ |  |  |
| --- | --- | --- | --- | --- |
| Temperature | **Helix** | **Beta** | **Turn** | **Random** |
| 20°C | 29.7 | 0 | 15.5 | 54.8 |
| 25°C | 31.6 | 0 | 16.6 | 51.8 |
| 30°C | 29.6 | 0 | 20.3 | 50.1 |
| 35°C | 28.5 | 0 | 21.8 | 49.7 |
| 40°C | 29.1 | 0 | 22.8 | 48.1 |
| 45°C | 31.1 | 0 | 22.5 | 46.4 |


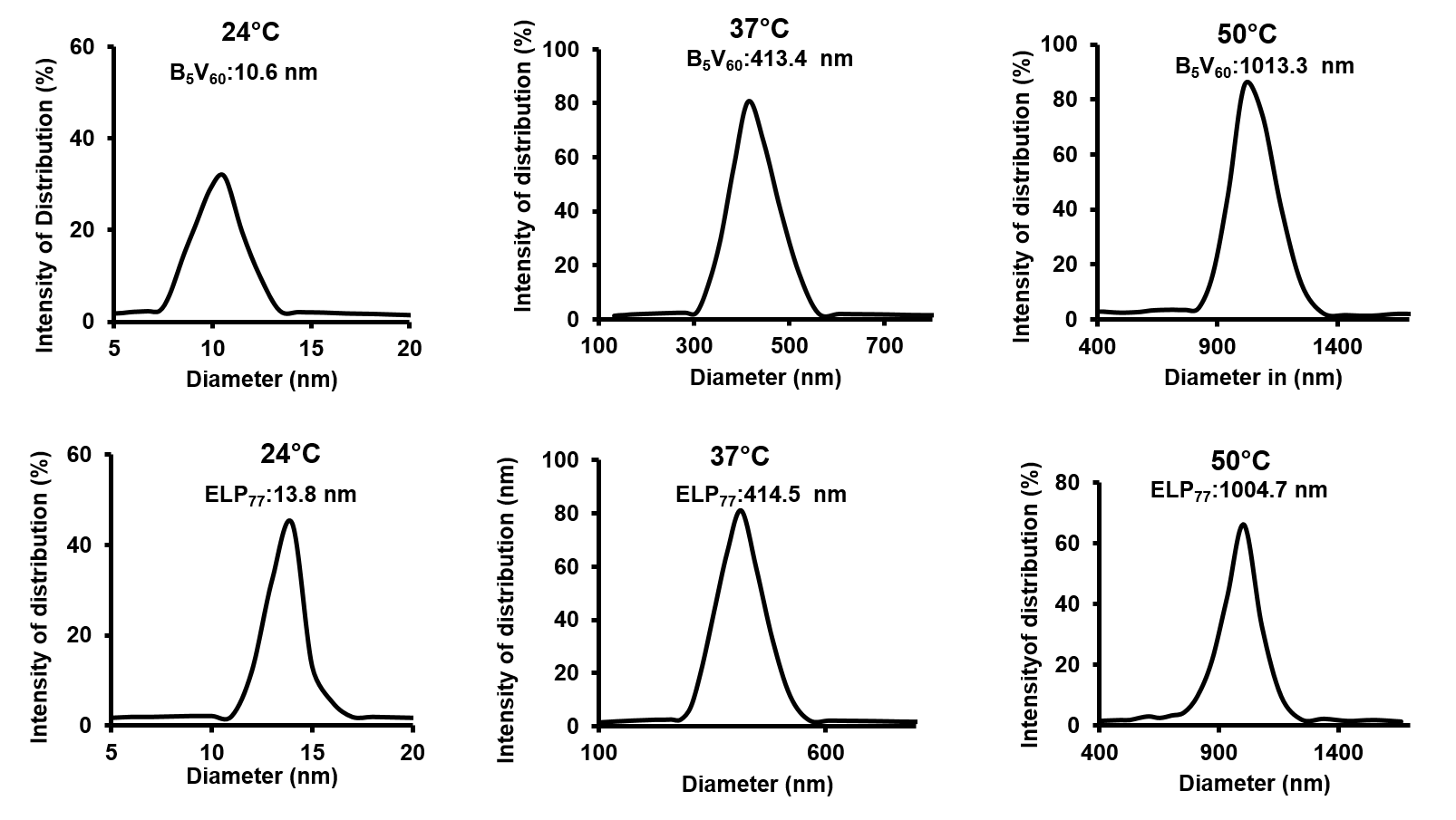


**Figure S3. Size determination by Dynamic Light Scattering (DLS)** The size was determined in 10 *µ*M **concentration at different temperatures (24, 37, and 50 °C) using DLS.** In consistence with turbidity profile the size of the polypeptides increase with increased in temperature. But at physiological temperature the size of the both ELP _77_ and B_5_V_60_ were 415.5 nm and 413.4 nm respectively.


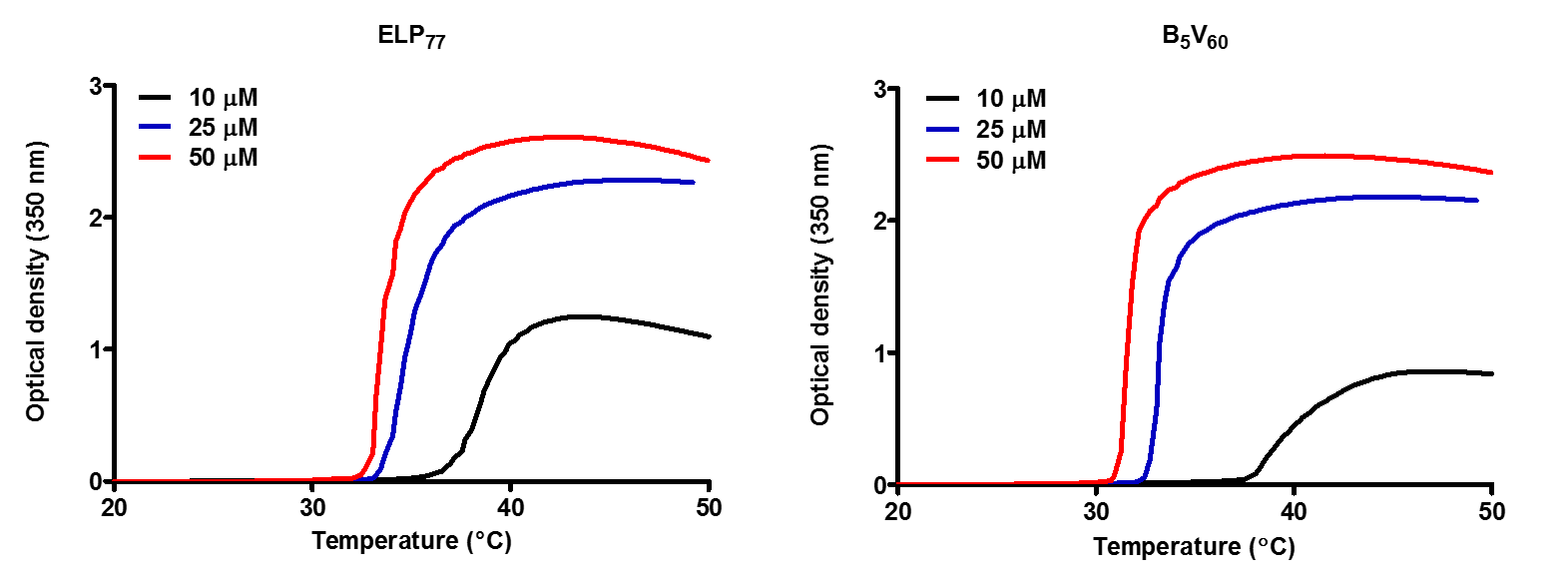


**Figure S4. Thermal characterization at different temperature.** The turbidity profile at different concentration were measured. Transition temperature of ELP_77_ were 38.67, 35.5, 33.8 **°C** at subsequent increased in concentration of 10, 25, 50 *µ*M, whereas the transition temperature of B_5_V_60_ were 39.77, 36.6, 34.8 **°C** at 10, 25, 50 *µ*M concentrations.


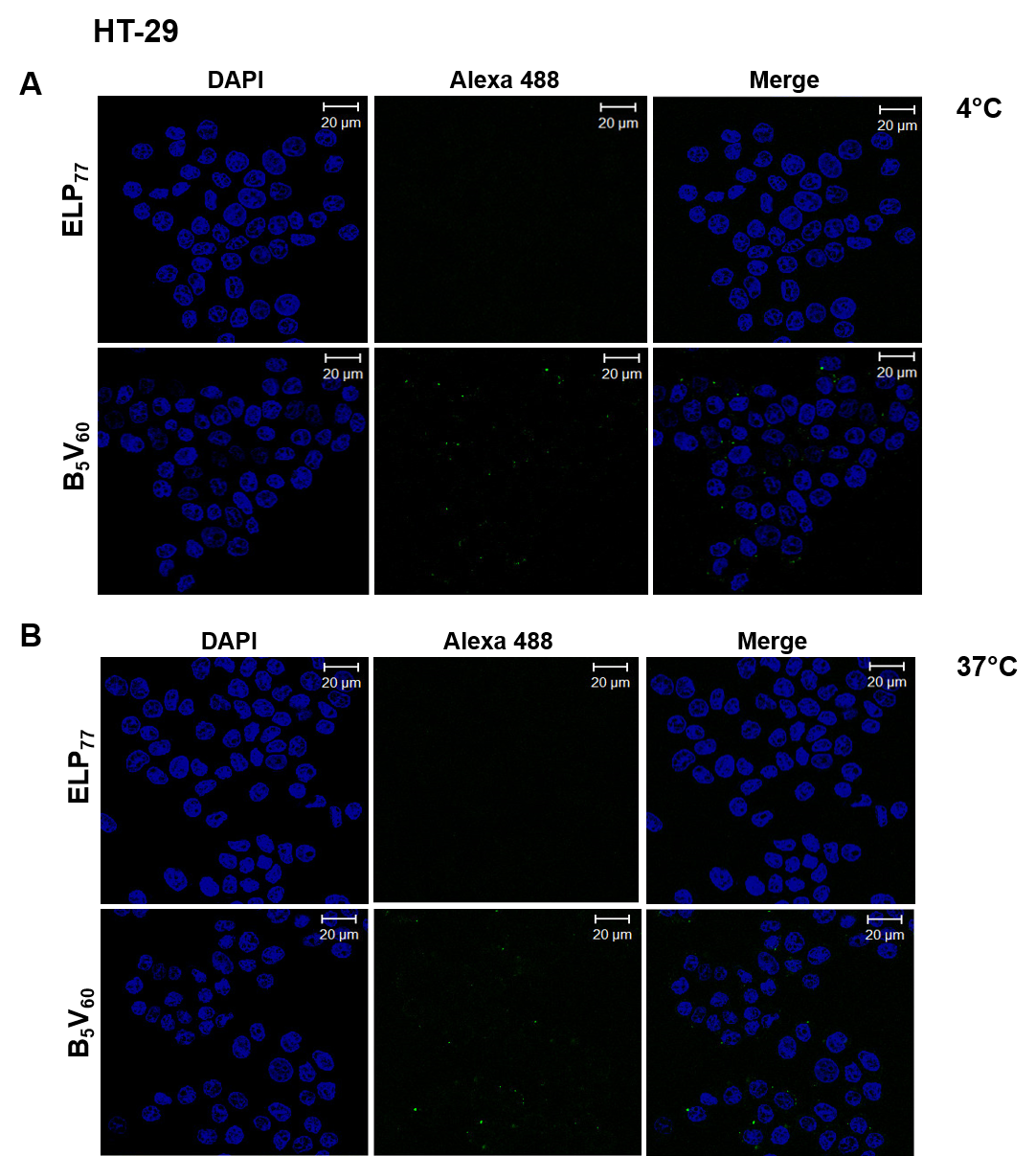


**Figure S5. Studies on cellular uptake *in vitro*.** Confocal images of HT-29 cancer cells treated with 10 *µ*M B_5_V_60_ or ELP_77_ at 4 °C (A) and 37 °C (B). Representative confocal images of three experiments (Scale bar 20 *µ*m).


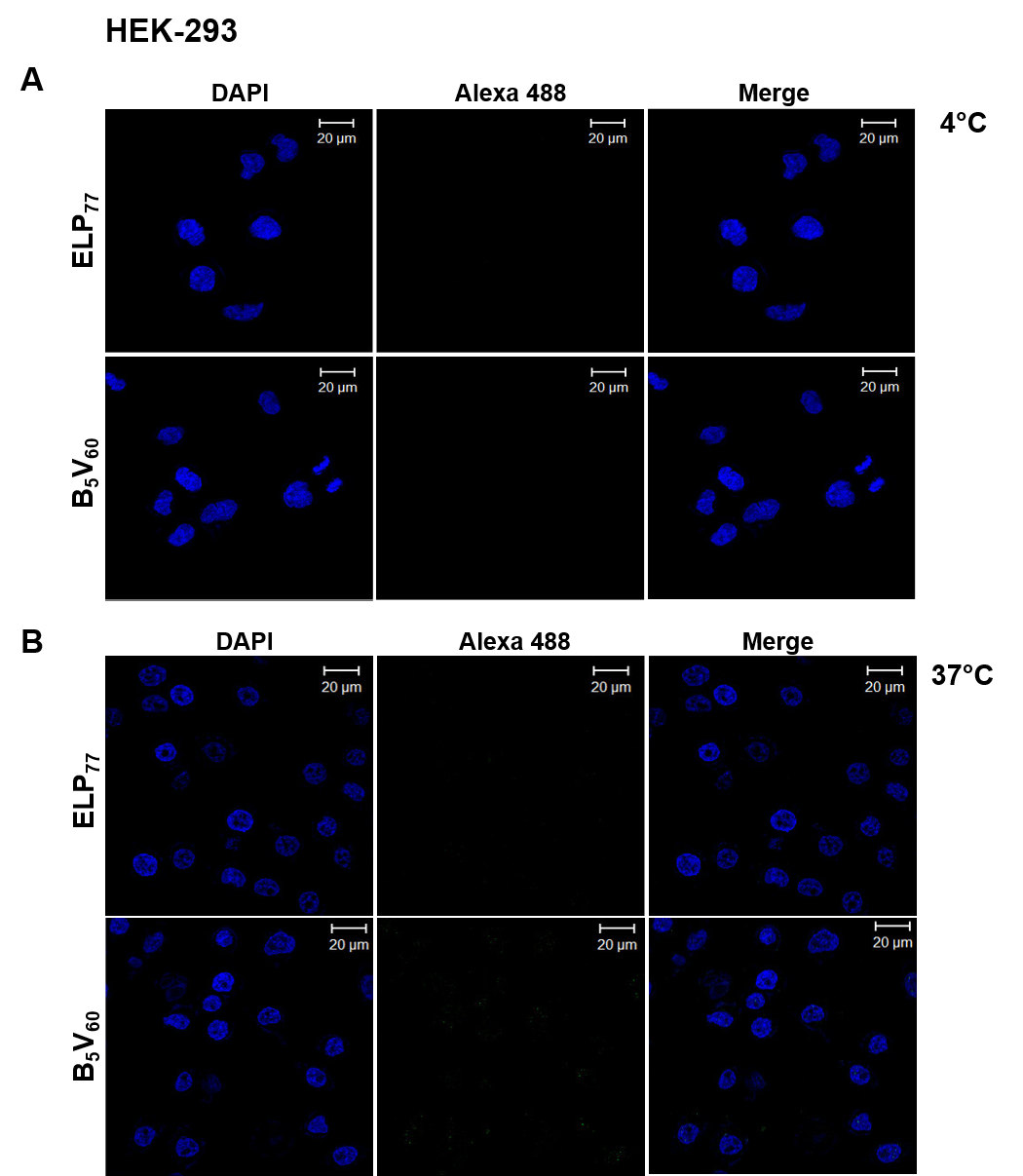


**Figure S6.** Confocal images of HEK-293 cells treated with 10 *µ*M ELP_77_ or B_5_V_60_ at 4 °C (A) and 37 °C (B). Scale bar 20 *µ*m.


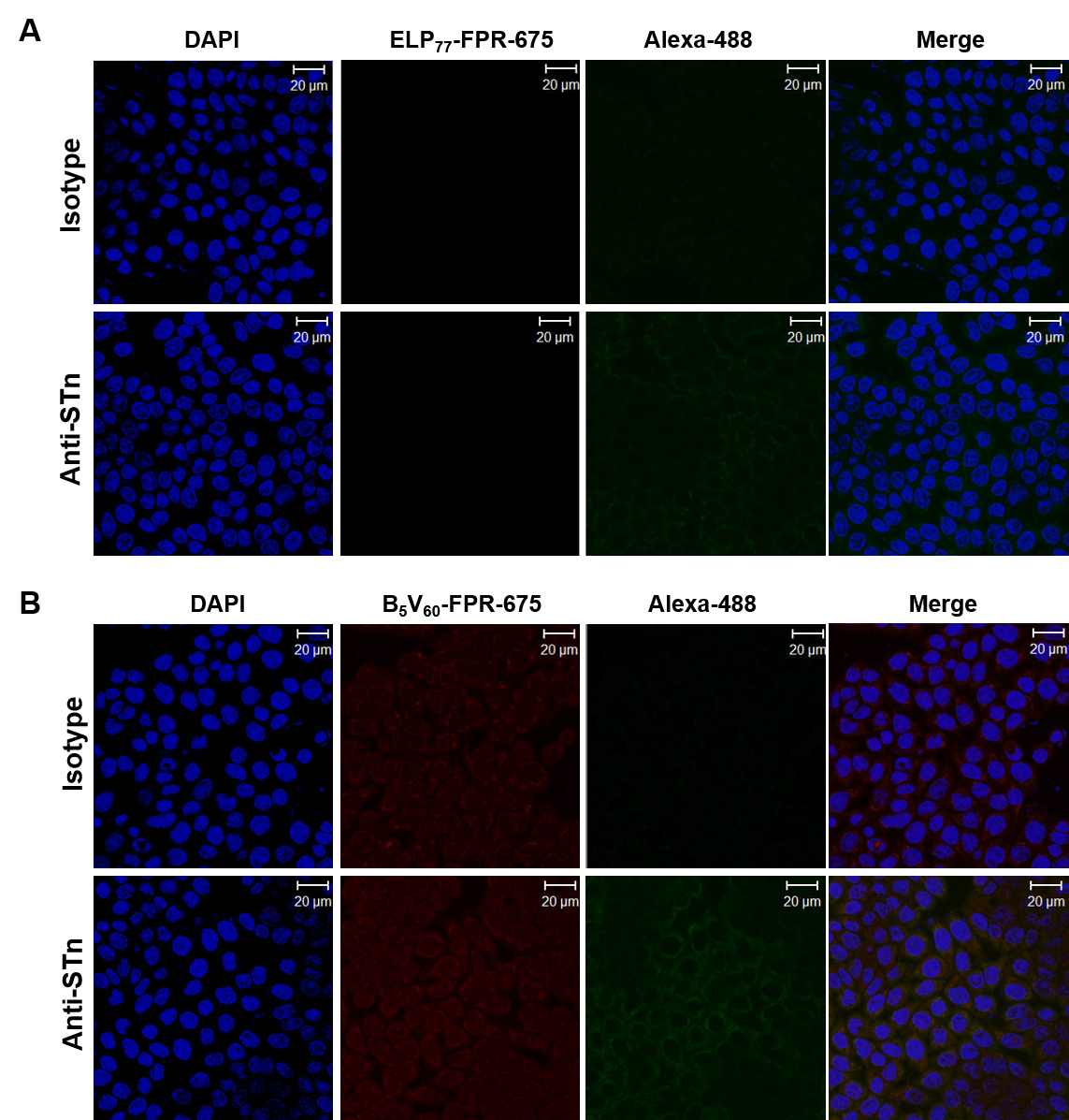


**Figure S7.** HT 29 cells were pre-incubated with anti-STn-Alexa 488 for 1 h at room temperature and further treated with 10 *µ*M B_5_V_60_ and ELP_77_ labeled with FPR-675. Co-localization of polymer and antibody was observed using confocal microscopy. Scale bar 20 *µ*m.


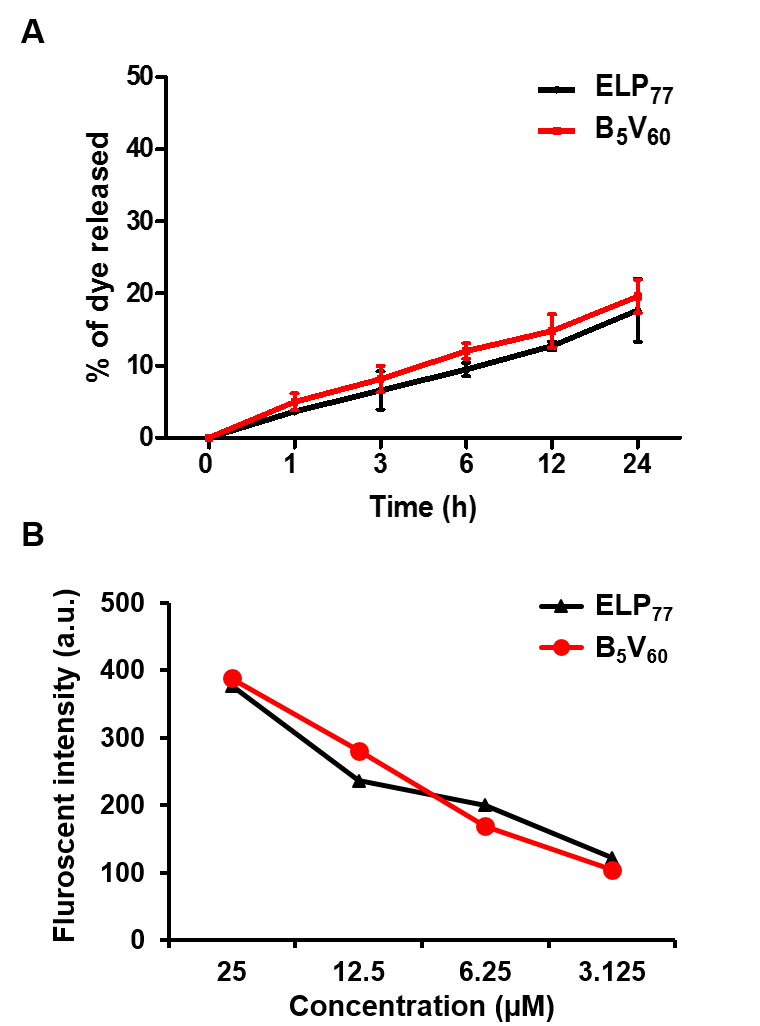


**Figure S8. A.** Labeling efficiency of FPR-675 conjugated with B_5_V_60_ and ELP_77_ was checked by incubation in mouse plasma for different time intervals. Post-ITC fluorescence intensities in pellet and supernatant were measured at an excitation wavelength of 675 nm and emission wavelength of 698 nm. The percentages of dye released at different time intervals were calculated. B. Prior to *in vivo* biodistribution study, fluorescence intensities of different concentrations of B_5_V_60_ and ELP_77_ were analyzed.


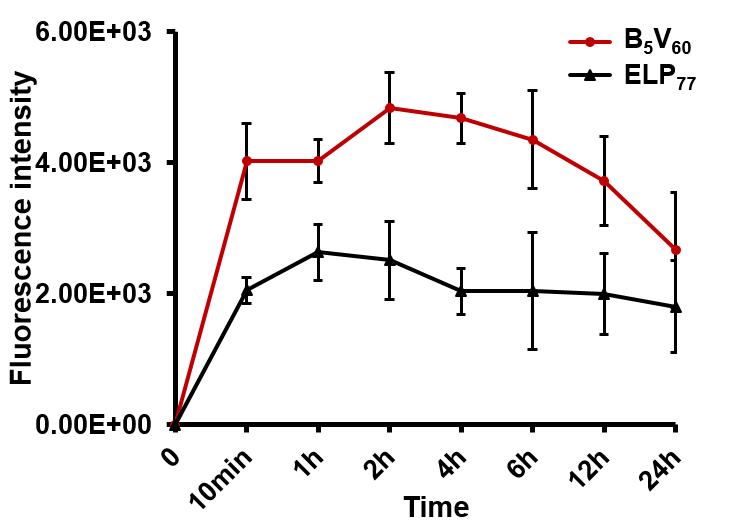


**Figure S9.** Fluorescence intensity of tumor tissue estimated from optix images taken at different time intervals (n=10).
